# Impacts of Metal–Support Interaction on Hydrogen Evolution Reaction of Cobalt-Nitride-Carbide Catalyst

**DOI:** 10.3389/fchem.2021.828964

**Published:** 2022-02-01

**Authors:** Xuan Zhang, Yu-An Li, Yaozhen Huang, Haiqiang Mu, Xiaofeng Gu, Feng Li, Zheng Wang, Jing Li

**Affiliations:** State Key Laboratory of High-Efficiency Utilization of Coal and Green Chemical Engineering, School of Chemistry and Chemical Engineering, Ningxia University, Yinchuan, China

**Keywords:** cobalt nanoparticles, green hydrogen, energy conversion, metal–support interaction, adsorption energy

## Abstract

Cobalt-nitride-carbide (Co-N-C) catalysts are promising cost-efficient transition metal catalysts for electrocatalytic hydrogen evolution, but few works investigate the metal–support interaction (MSI) effect on hydrogen evolution reaction (HER) performance. Herein, efficient Co-N-C_X_ catalysts with controllable MSI between encapsulated Co nanoparticles and nitrogen-doped graphitic carbon nanosheets were synthesized via a facile organic–inorganic hybridization method. Results demonstrate that the Co-N-C_0.025M_ catalyst with the coexistence of single-atom Co sites and Co nanoparticles prepared by 0.025 M cobalt nitrate shows excellent HER performance, achieving a low overpotential of 145 mV to reach 10 mA cm^−2^ in 0.5 M sulfuric acid, which is mainly because the optimal MSI, which leads to a moderate hydrogen adsorption energy and improved electroactive sites, not only facilitates the charge transfer to improve the HER kinetics, but also improves the durability of the catalyst by Co-N bond anchoring and encapsulation of active Co species. This work provides guidance to further reveal the influence of MSI on their catalytic activity.

## Introduction

Hydrogen energy is believed to be an ideal energy source to counter climate-related environmental degradation and mitigate energy crisis thanks to its renewability, high energy density, and the absence of greenhouse gas emissions (Zhu et al., 2020). Additionally, hydrogen evolution reaction (HER) through electrolytic water splitting has been considered as an efficient approach to transfer intermittent energy sources such as solar or wind power to stable hydrogen energy (Li et al., 2020a). The Pt-based catalysts are well known as the most ideal electrocatalytic materials for HER, but Pt resources are scarce and expensive, which hinder its large-scale industrialization (Liu et al., 2019). Therefore, it is indispensable to find non-precious metal alternatives with abundant resources and outstanding catalytic activity for HER.

Transition metal compounds, such as metal oxides (CoO_x_, FeO_x_, and CuO_x_) (Ling et al., 2017; Suryanto et al., 2019; Zhang et al., 2021), sulfides (MoS_x_ and CuS_x_) (Guo et al., 2019a; Aslan et al., 2019; Yang et al., 2021), phosphides (CoP_x_ and WP_x_) (Du et al., 2018; Liu et al., 2021; Zhao et al., 2021), nitrides (MoN_x_ and CoN_x_) (Jin et al., 2018; Peng et al., 2019; Shu et al., 2020), carbides (Ni_3_C and Mo_2_C) (Li et al., 2016; Gao et al., 2019; Lu et al., 2019; Ma et al., 2020), and metal-nitride-carbide (M-N-C, M = Fe, Co, Ni, etc.) (Liu et al., 2017; Roy et al., 2018; Jin et al., 2019; Shi et al., 2020), have gradually attracted attention in electrocatalytic hydrogen production applications (Dinh et al., 2019). Among these transition metal electrocatalysts, the Co-based electrocatalysts have been proposed as the ideal alternatives for cost-efficient and highly active HER owing to their extensive availability, high catalytic performance, and being environmental friendly (Zhang et al., 2020).

In recent years, Co-N-C has attracted growing interest as a highly efficient catalyst for HER (Deng et al., 2019; Sa et al., 2019). Usually, single-atom Co sites are regarded as the most active sites (Sun et al., 2018), and when the Co-N-C electrocatalysts are synthesized, strong acid etching is used to remove Co nanoparticles (NPs) agglomerated during thermal polymerization. For example, Sun et al. (2018) compared the HER performance of as-prepared cobalt based catalyst with single-atom Co sites encapsulated in hierarchically ordered porous nitrogen-doped carbon (Co-SAS/HOPNC) with the acid-treated Co-NPs/HOPNC electrocatalyst to confirm that atomically dispersed Co sites contribute to the enhanced HER activity, and the overpotential of Co-SAS/HOPNC catalyst was 137 mV in 0.5 M sulfuric acid at the current density of 10 mA cm^−2^. However, numerous studies demonstrate that hydrogen evolution activity not only depended on single-atom Co sites, but also was affected by the interaction between embedded metallic cobalt particles and nitrogen-doped carbon supports (Wang et al., 2014; Guo et al., 2019b; Jia et al., 2019; van Deelen et al., 2019; Zhang et al., 2019; Yang et al., 2020). Benefiting from the strong synergy between Co NPs and carbon supports, the hybrid Co-N-C catalysts showed excellent HER activity. For instance, Chen et al. (2018) fabricated an efficient electrocatalyst with ultrafine Co NPs embedded in nitrogen-doped carbon nanotube grafted graphene nanosheets, and the as-prepared composite catalyst exhibited remarkable HER performance to reach 10 mA cm^−2^ at a low overpotential of 87 mV in 0.5 M H_2_SO_4_. Additionally, Lyu et al. (2019) prepared efficient Co-N-C catalysts with a hybrid structure comprising Co-N species and Co NPs embedded in nitrogen-doped carbon shell, which could achieve an overpotential of 180 mV to reach 10 mA cm^−2^ in 1.0 M KOH. Furthermore, Du et al. (2020a) synthesized a uniform Co NP (about 7 nm in diameter) embedded in nitrogen-doped carbon that exhibited high HER activity with a stabilized overpotential of 180 mV at the current density of 10 mA cm^−2^ in sulfuric acid medium. However, the synthesis procedures of these Co NPs-based Co-N-C catalysts are usually complex and involve expensive modulator or template agents, which is not appropriate for the extensive commercial application.

Metal–support interaction (MSI) is of great importance for heterogeneous catalysis, which is widely exploited as a strategy to improve the catalytic activity, due to the synergy effect on chemical bonding and electron transition, where the chemical bonding at the interfacial provides a bridge for the electron transition between the metal and support, leading to a change of the charge distribution on the metal surface and further on the adsorption energy. but a full investigation of the nature of MSI has not been achieved on the HER electrocatalysts.

Herein, a facile one-pot organic–inorganic hybridization method was employed to synthesize Co-N-C hybrid catalysts with single-atom Co sites and encapsulated Co NPs for HER, which act as a model catalyst to investigate the influence of MSI on the HER activity. The cobalt nitrate was employed as the metal precursor, and glucose and dicyandiamide were applied as the carbon and nitrogen precursors, respectively. The concentration of cobalt nitrate was regulated to control the diameter of the Co NPs, so as to adjust the interaction between encapsulated Co NPs and nitrogen-doped carbon supports to obtain optimized hydrogen evolution activity in acid medium. Finally, the Co-N-C_0.025M_ catalyst demonstrated the best HER performance, which could achieve a low overpotential of 145 mV to reach 10 mA cm^−2^ in 0.5 M H_2_SO_4_. The remarkable hydrogen evolution activity and good durability were attributable to the strong synergistic effects between single-atom Co sites and embedded Co NPs that had the suitable interaction with surrounding nitrogen-doped carbon supports.

## Experimental Section

### Materials

Glucose (14431-43-7, 98%), dicyandiamide (461-58-5, 99%), Co (NO_3_)_2_∙6H_2_O (10026-22-9, 99.99% metals basis), Fe(NO_3_)_3_∙9H_2_O (7782-61-8, 99.999% metals basis), Cu (NO_3_)_2_∙3H_2_O (10031-43-3, 99.99% metals basis), and (NH_4_)_6_H_2_W_12_O_40_∙xH_2_O (12333-11-8, 99.5% metals basis) were purchased from Aladdin Chemical Reagent Co., Ltd. H_2_SO_4_ (7664-93-9, AR 95.0%–98.0%), HCl (7647-01-0, AR 36.0%–38.0%), and C_2_H_5_OH (64-17-5, AR ≥ 99.5%) were purchased from Sinopharm Chemical Reagent Co., Ltd. All aqueous solutions were prepared with a Milli Q water purification system (18.2 MΩ cm), and all the reagents and solvents employed were commercially available and used as received without further purification.

### Synthesis of Co-N-CX

In general, 0.25 g glucose (14431-43-7, 98%) and 5 g dicyandiamide (461-58-5, 99%) were dissolved in 200 ml of deionized water, and 2 ml of Co (NO_3_)_2_∙6H_2_O (10026-22-9, 99.99% metals basis) solution of certain concentration was added dropwise to the above solution with vigorous stirring. After stirring for 2 h, the solvent was evaporated under reduced pressure, and the obtained solid was carbonized at 900°C for 2 h under an Argon atmosphere (ramp rate = 3°C min^−1^). The products were abbreviated as Co-N-C_X_, where X was the concentration of Co (NO_3_)_2_∙6H_2_O solutions. Additionally, N-C was prepared for comparison, using the same route as for Co-N-C_X_, except for the addition of Co (NO_3_)_2_∙6H_2_O solution.

### Synthesis of Acid-Treated Co-N-C_X_

Twenty-five milligrams of each Co-N-C_X_ catalyst was treated by 250 ml of 1 M HCl for 8 h at 80°C with reflux, respectively. Then, the samples were vacuum-dried at 60°C overnight after washing with deionized water, and the final products were abbreviated as H-Co-N-C_X_.

### Synthesis of M-N-C_X_

M-N-C_X_ samples were synthesized using the same method as for Co-N-C_X_, where M was Mo, Fe, W, Ni, and Cu rather than Co.

### Electrochemical Measurements

The electrochemical measurements were conducted on a CHI 760E electrochemical workstation (Shanghai Chenhua Co., Ltd., Shanghai, China) with a standard three-electrode system. A graphite electrode was used as the counter electrode, and an Ag/AgCl electrode (KCl-saturated) was employed as the reference electrode. A glassy carbon rotating disk electrode (RDE) (Model 636A, Princeton Applied Research, Ametek Advanced Measurement Technology Inc.) with coated electrocatalysts was used as the working electrode, which was prepared as follows: 2 mg of catalyst and 500 μl of 0.5% Nafion solution were homogenously dispersed under ultrasound conditions in 1.5 ml of ethanol–water solution at room temperature (the volume ratio between ethanol and deionized water was 2:1). Then, 30 μl of catalyst ink was dropped onto the polished glassy carbon surface (4 mm in diameter), leading to a catalyst loading of 0.24 mg cm^−2^. The HER tests were carried out with RDE at a rotation rate of 2,000 rpm, and linear sweep voltammetry (LSV) measurements were recorded at a scan rate of 10 mV s^−1^ in 0.5 M H_2_SO_4_ solution. Electrochemical impedance spectroscopy (EIS) was performed over a frequency range from 0.1 MHz to 0.1 Hz with an amplitude of 5 mV. All the potentials in this study were iR corrected and converted to the reversible hydrogen electrode (RHE). The Ag/AgCl electrode was calibrated with respect to RHE, using Pt as working and counter electrodes, purged with high pure hydrogen gas during the measurement (Supplementary Figure S1) (Mukherjee et al., 2016). The calibration value was: E_*vs*. RHE_ = E_*vs.*_ _Ag/AgCl_ + 0.059pH + 0.285 V.

## Results and Discussion

**SCHEME 1:**
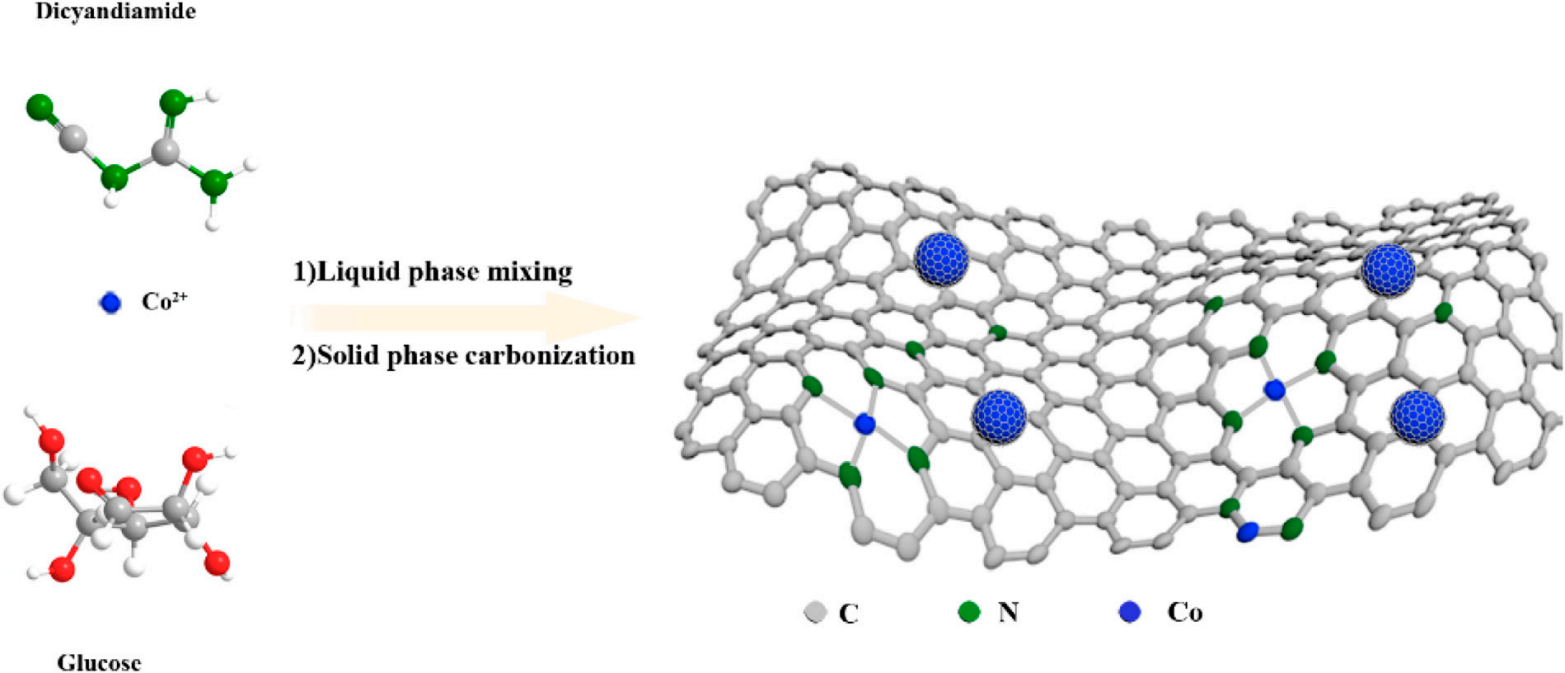
The schematic illustration for the preparation of Co-N-C_X_.

The Co-N-C_X_ catalysts were synthesized as follows (as shown in Scheme 1): (1) The precursor solution was synthesized via a one-pot reaction by just mixing dicyandiamide, glucose, and cobalt nitrate solution with concentration X. (2) The precursor solution was dried well under reduced pressure and the remaining powder was then carbonized at high temperature under an Argon atmosphere to obtain Co-N-C_X_. In addition, the Co-N-C_X_ catalysts were further heated under reflux in hydrochloric acid to gain H-Co-N-C_X_ for comparison. This method was also used to prepare other M-N-C_X_; however, the performance of the Co-N-C_0.025M_ catalyst was significantly better than other M-N-C_0.025M_ catalysts, and the Co-N-C_0.025M_ prepared at 900°C was found to yield the best HER activity, eventually (as shown in Supplementary Figures S2, S3).

**FIGURE 1:**
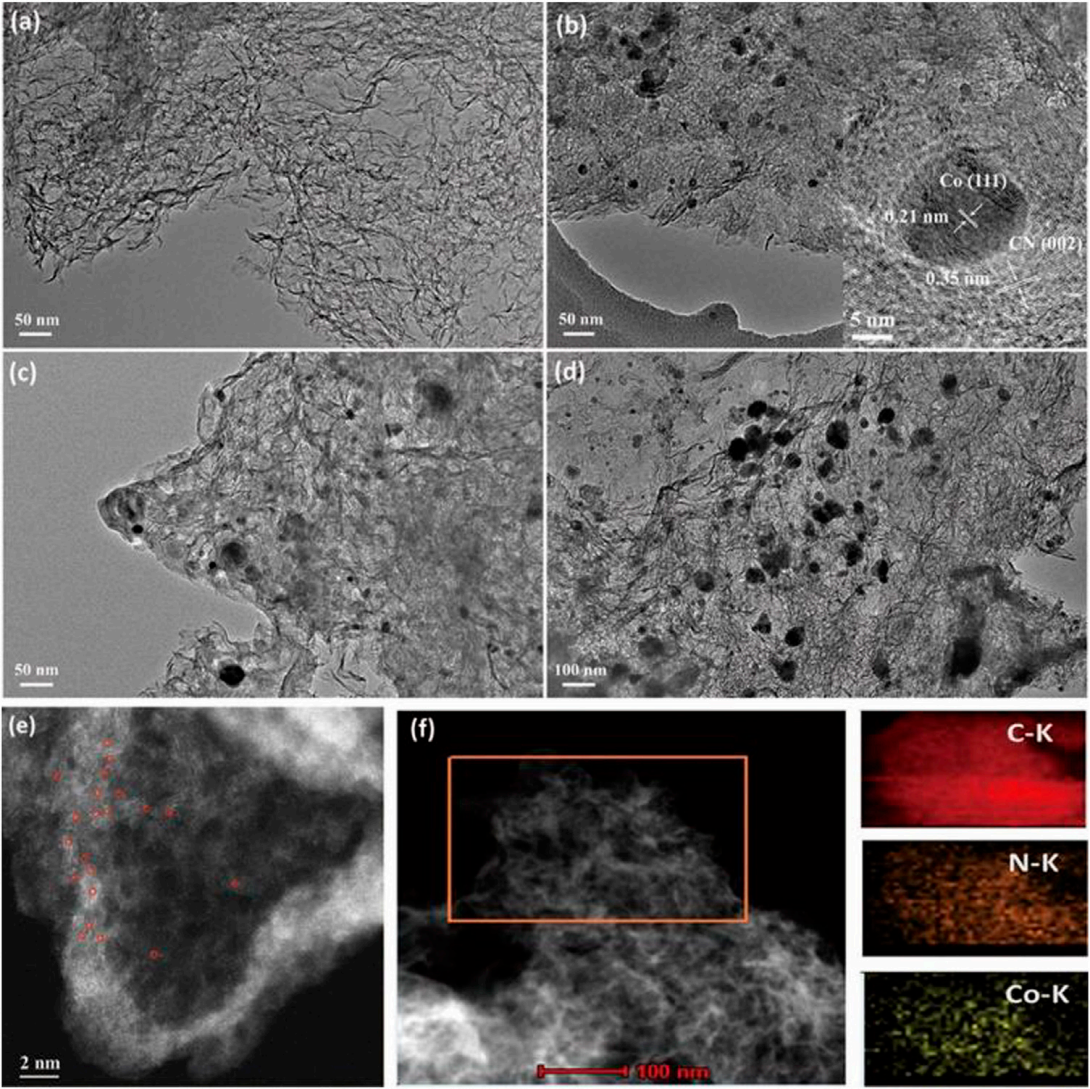
TEM images of the Co-N-C_X_ catalysts: **(A)** Co-N-C_0.0125M_, **(B)** Co-N-C_0.025M_ (Inset: HRTEM image of Co-N-C_0.025M_). **(C)** Co-N-C_0.05M_ and **(D)** Co-N-C_0.075M_. **(E)** HAADF-STEM image of Co-N-C_0.0125M_. **(F)** STEM image and corresponding element mapping of Co-N-C_0.025M_.

The morphologies of the Co-N-C_X_ catalysts were characterized through transmission electron microscopy (TEM) linked to an x-ray energy dispersive spectrometer (EDS). The TEM images (Figures 1A–D) showed that all the Co-N-C_X_ catalysts possessed transparent and wrinkled characteristics, regarded as the structural features of ultrathin graphene-like carbon nanosheets (Wang et al., 2019). Although it was not very obvious, there were few small Co NPs presented in Co-N-C_0.0125M_ (Figure 1A). Aberration-corrected high-angle annular dark-field scanning transmission electron microscopy (HAADF-STEM) was applied to further investigate the structural details of Co-N-C_0.0125M_, and single-atom Co sites, which were distinguished as brighter spots in Figure 1E, were noted homogeneously dispersed throughout the carbon supports in Co-N-C_0.0125M_. In comparison with Co-N-C_0.0125M_, spherical Co NPs were easily observed in Co-N-C_0.025M_, Co-N-C_0.05M_, and Co-N-C_0.075M_, and the particle size gradually increased from about 30 to 70 nm (Figures 1B–D) with the increase of the cobalt precursor concentration. This phenomenon revealed that the concentration of cobalt precursor used in the synthesis could be regulated to adjust the diameter of the Co NPs to obtain a hybrid catalyst with coexistence of single-atom Co sites and Co NPs. In addition, the HRTEM image (inset of Figure 1B) showed that Co nanoparticle was tightly encapsulated with several layers of carbon nanosheets, and the lattice fringe of Co nanoparticle was 0.21 nm, corresponding to the (111) crystal plane of β-Co phase. Moreover, the lattice fringe of surrounding carbon nanosheets was 0.35 nm, which was slightly larger than pure graphitic carbon, on account of the successful doping of nitrogen into the carbon matrix (Jia et al., 2019). Element mapping was performed to analyze the element distribution, and the elements Co, N, and C were found distributed uniformly on the carbon supports (Figure 1F). Corresponding to the TEM image, the nanosheet structure of the Co-N-C_0.025M_ sample could also be observed in the AFM image (Supplementary Figure S4), and the thickness of the carbon nanosheet was about 3.42 nm. Thus, the as-prepared Co-N-C_X_ catalysts had a hybrid structure comprising single-atom Co sites and embedded Co NPs, while Co NPs and the nitrogen-doped carbon supports were in intimate contact. The presence of encapsulated Co NPs would affect the features of the surrounding carbon supports by altering the electron density. This might boost the electron transfer from carbon supports to Co NPs during the HER processes and was helpful in promoting high catalytic performance.

**FIGURE 2:**
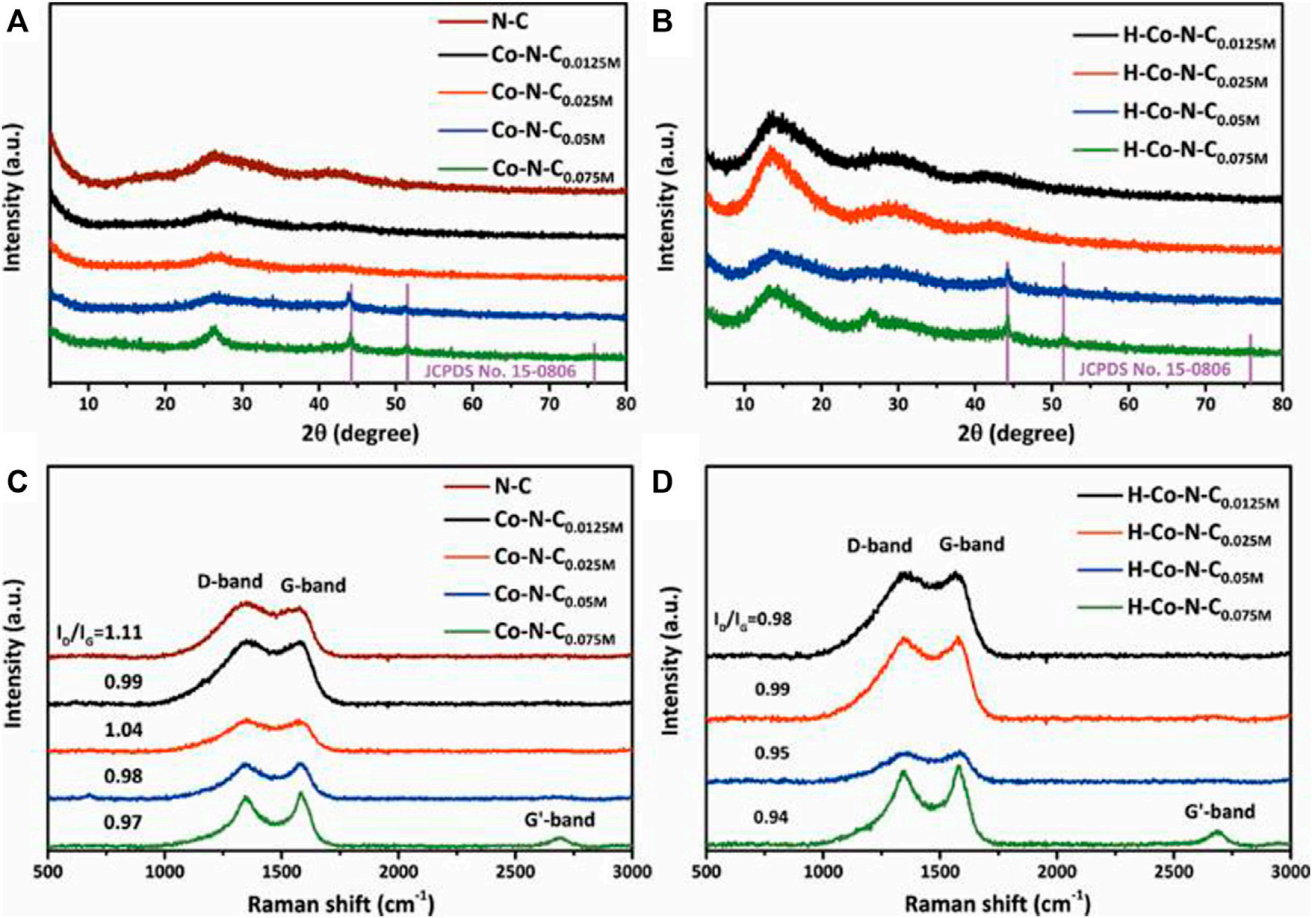
XRD patterns of **(A)** N-C, Co-N-C_X_ and **(B)** H-Co-N-C_X_, and Raman spectrum of **(C)** N-C, Co-N-C_X_, and **(D)** H-Co-N-C_X_.

The crystal phases of the as-prepared catalysts were analyzed by x-ray powder diffraction (XRD), and the XRD patterns of N-C, Co-N-C_X_, and H-Co-N-C_X_ are presented in Figures 2A,B. As shown in Figure 2A, the N-C sample displayed two broadening diffraction peaks around 26.5° and 43.3°, which corresponded to the (002) and (100) lattice planes of graphitic carbon, respectively (Chen et al., 2018). As expected, for the Co-N-C_0.0125M_ and Co-N-C_0.025M_, there were two diffraction planes corresponding to graphitic carbon, but no peaks related to the crystalline cobalt. However, for the Co-N-C_0.05M_ and Co-N-C_0.075M_, besides the broadened peaks of graphitic carbon, additional sharp peaks located at 44.2° [Co (111)] and 51.5° [Co (200)], which were related to the β-Co phase (JCPDS No. 15-0806), were observed. Interestingly, a broadening diffraction peak around 13.3° appeared after acid treatment for each H-Co-N-C_X_ sample, which corresponded to the typical interplanar structural packing of the graphitic-like carbon nitride and indicated that acid treatment could destroy the interlayer stacking of the graphite-like structure, making nitrogen-doped graphitic carbon become more like the planar graphene structure with much sp2 hybridized carbon (Tian et al., 2013; Wu et al., 2015). The structural changes of the nitrogen-doped carbon supports might lead to changes of the MSI, and the Co NPs that had weak interaction with the carbon supports might be etched off by acid, while the well-encapsulated Co NPs that had strong MSI were not affected, since the diffraction peaks of crystalline cobalt did not change significantly for both H-Co-N-C_0.05M_ and H-Co-N-C_0.075M_ (Figure 2B).

To analyze the structure of nitrogen-doped carbon nanosheets, Raman spectroscopy was employed. As shown in Figure 2C, the two strong peaks at about 1,349 and 1,590 cm^−1^ corresponded to the D and G bands, respectively, where the D band was related to disordered carbon structure and the G band was representative features of in-plane vibrations of sp2 hybridized carbon (Wang et al., 2014; Guo et al., 2019b). The relative intensity ratios of D/G (I_D_/I_G_) calculated from the peak intensity indicated the defect level and degree of graphitization of carbon structure. Figure 2C demonstrates that the I_D_/I_G_ value for N-C was 1.11, and the I_D_/I_G_ value of Co-N-C_0.025M_ was 1.04, which was higher than those of Co-N-C_0.0125M_ (0.99), Co-N-C_0.05M_ (0.98), and Co-N-C_0.075M_ (0.97). It suggested that Co-N-C_0.075M_ had a higher degree of graphitization, which was consistent with the XRD results in which Co-N-C_0.075M_ exhibited a sharper diffraction peak of graphitic carbon (Figure 2A). Compared with N-C, the addition of Co species could affect the *in situ* nitrogen doping and carbonization process, gaining higher degree of graphitization accordingly (Jia et al., 2019). In addition, when the concentration of cobalt precursor increased, the cobalt NPs agglomerated to form large particles, which would change the interaction between Co species and graphitic carbon supports; thus, I_D_/I_G_ values of the corresponding Co-N-C_X_ catalyst showed a decreased trend, except for Co-N-C_0.025M_. This exception indicated that Co-N-C_0.025M_ had the highest level of nitrogen-doped sites, which enabled optimal MSI among the Co-N-C_X_ catalysts. This feature could have a great contribution to the HER performance. Furthermore, the I_D_/I_G_ values of the acid-treated H-Co-N-C_X_ samples were lower than that of the corresponding Co-N-C_X_ (Figure 2D), demonstrating again that acid treatment would destroy the graphite-like stacking and recover the in-planar structure (Wu et al., 2015).

**FIGURE 3:**
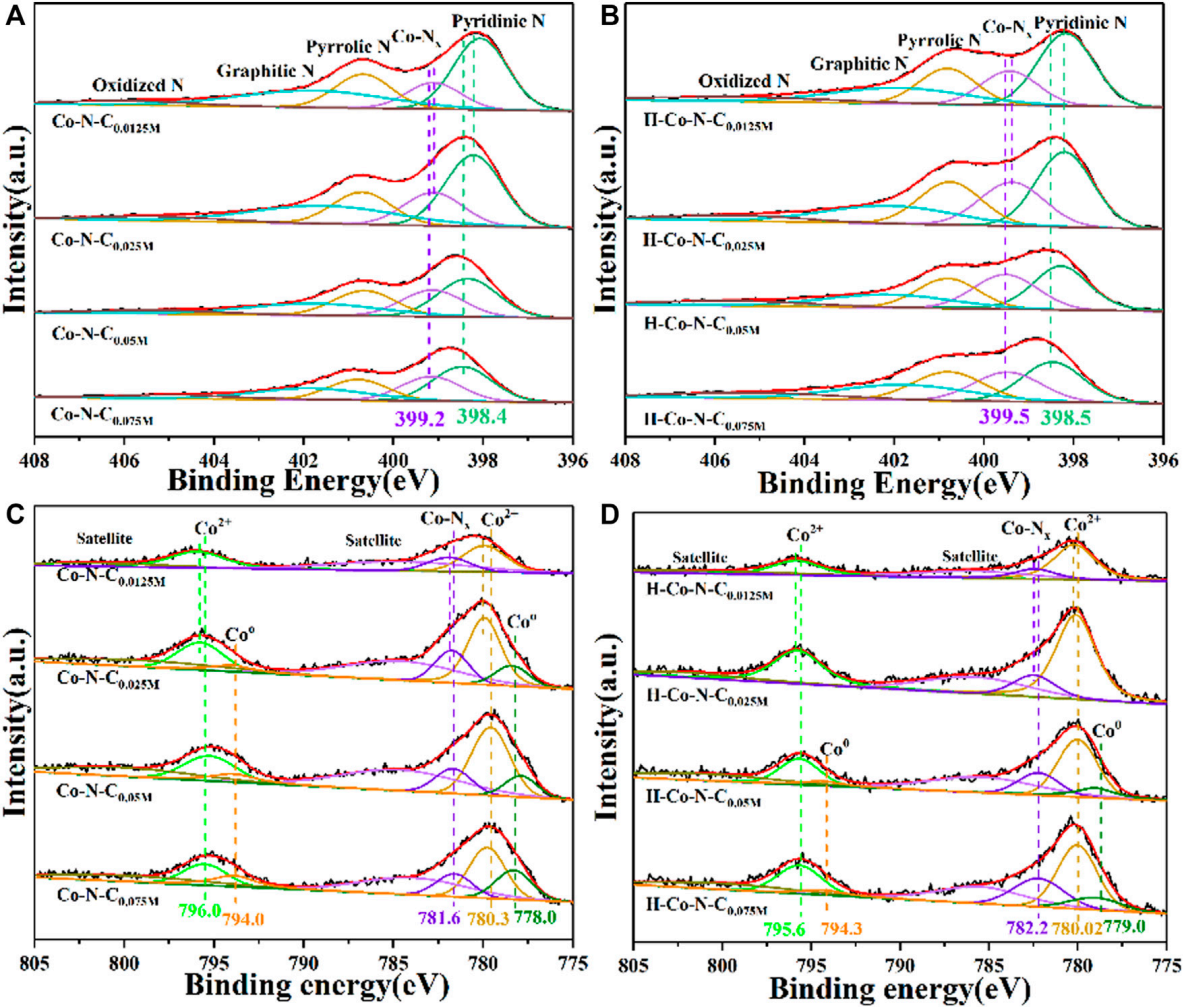
High-resolution N 1s spectra of **(A)** Co-N-C_X_ and **(B)** H-Co-N-C_X_, and high-resolution Co 2 p spectra of **(C)** Co-N-C_X_ and **(D)** H-Co-N-C_X_.

The surface elemental composition and chemical states of the Co-N-C_X_ and H-Co-N-C_X_ samples were verified by x-ray photoelectron spectroscopy (XPS) (Supplementary Figure S5). The high-resolution N 1s spectrum (Figure 3A) of Co-N-C_0.0125M_ indicated that pyridinic N, Co-N_x_, pyrrolic N, graphitic N, and oxidized N were located respectively at 398.4, 399.2, 400.7, 401.8, and 405.5 eV. Compared with Co-N-C_0.075M_, there were slight negative displacements of Co-N_x_ when the concentration of cobalt precursor decreased, and Co-N-C_0.0125M_ showed the lowest binding energy of Co-N_x_. This is mainly due to the different electronegativity causing an electron transfer from Co to N; thus the binding energy shift can be used as an indicator to the MSI, because the strong MSI always leads to an obviously electronic environment change of Co-N_x_, and the same trend can be observed for all H-Co-N-C_X_ after acid treatment (Figure 3B). Curve fitting of the high-resolution Co 2p peak spectrum of Co-N-C_X_ and H-Co-N-C_X_ are shown in Figures 3C,D, respectively. For Co-N-C_0.0125M_, two main peaks appeared at 780.0 and 795.8 eV, demonstrating that Co was mainly in divalent Co state, which might be due to the strong interaction between the Co species and nitrogen-doped carbon nanosheets (Song et al., 2017; Guo et al., 2019b). Moreover, the peak at 781.7 eV was ascribed to Co-N_x_ species. Low-intensity peak located at 778.0 eV could be attributed to metallic Co NPs. A minor positive shift of Co^0^, Co^2+^, and Co-N_x_ would be observed when compared Co-N-C_0.075M_ with other Co-N-C_X_. When the concentration of cobalt precursor decreased, the binding energy of Co-N_x_ gradually shifts to the positive direction and the fitting peak area of metallic Co decreases obviously, and declines in proportion, indicating that more metallic Co species are encapsulated to weaken the MSI. Offset with moderate binding energy of Co-N-C_0.025M_ illustrated that there were electron transfer processes between Co species and the nitrogen-doped carbon supports, and the unique MSI of Co-N-C_0.025M_ might lead to outstanding catalytic performance for HER (Hernandez Mejia et al., 2018). Compared with Co-N-C_X_, there were obviously negative offset of Co^0^ and little positive deviance of Co-N_x_ for all H-Co-N-C_X_; these changes demonstrated that the interaction between Co species and surrounding nitrogen-doped carbon supports might be changed by acid etching, causing a decrease in HER catalytic performance.

**FIGURE 4:**
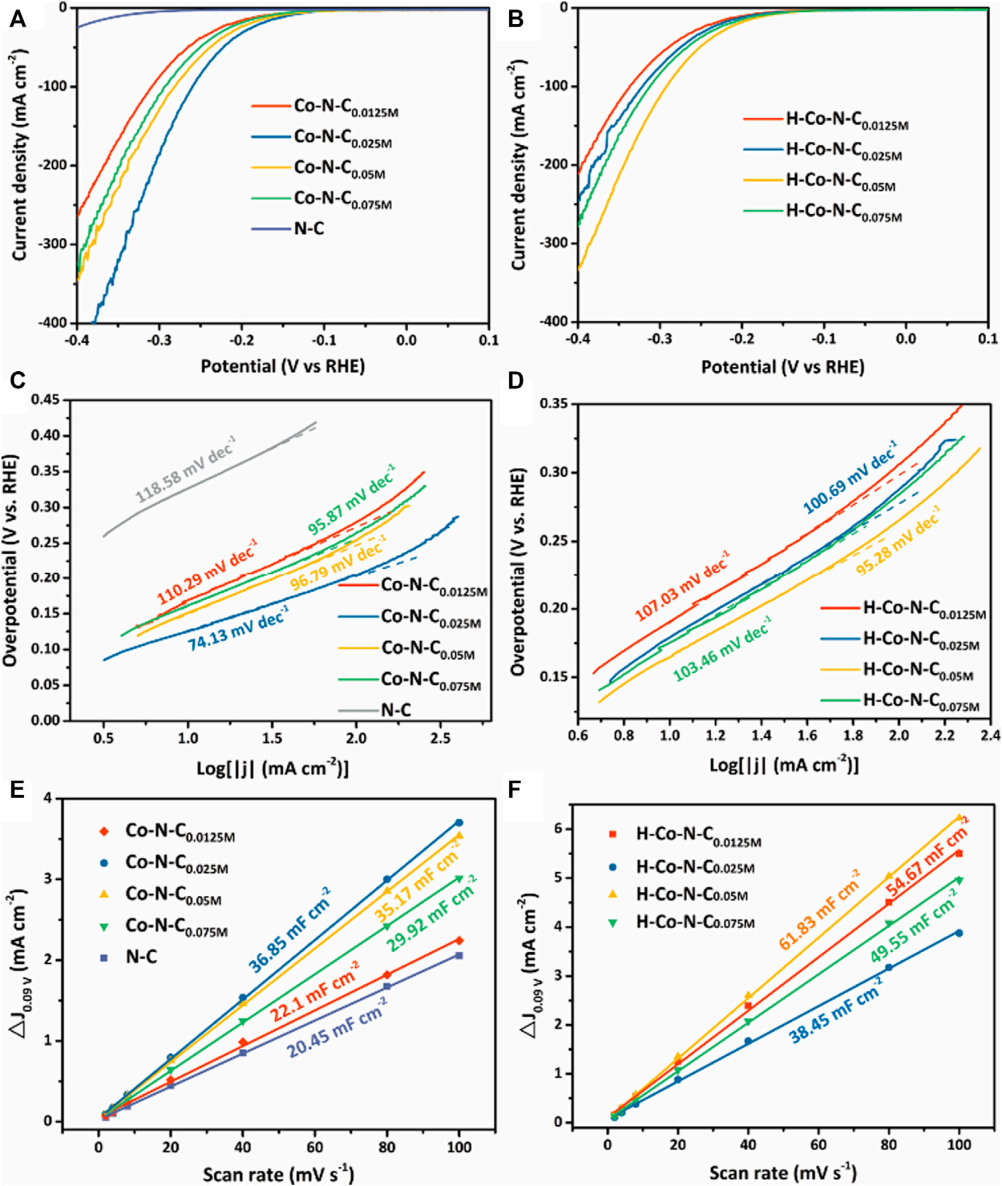
LSV curves of **(A)** Co-N-C_X_ and **(B)** H-Co-N-C_X_ in 0.5 M H_2_SO_4_. Tafel plots of **(C)** Co-N-C_X_ and **(D)** H-Co-N-C_X_ in 0.5 M H_2_SO_4_. C_dl_ values of **(E)** Co-N-C_X_ and **(F)** H-Co-N-C_X_.

The electrocatalytic HER performance of the as-synthesized catalysts was investigated using linear sweep voltammetry (LSV) in 0.5 M H_2_SO_4_. The polarization curves of Co-N-C_X_ and H-Co-N-C_X_ were compared in Figures 4A,B, respectively. For the Co-N-C_X_ catalysts, the overpotential of Co-N-C_0.0125M_, Co-N-C_0.025M_, Co-N-C_0.05M_, and Co-N-C_0.075M_ at a current density of 10 mA cm^−2^ was 176, 145, 158, and 170 mV, respectively. In the series of H-Co-N-C_X_ samples, H-Co-N-C_0.05M_ possessed much lower overpotential (172 mV) than those of H-Co-N-C_0.0125M_ (198 mV), H-Co-N-C_0.025M_ (191 mV), and H-Co-N-C_0.075M_ (182 mV). In addition, potassium thiocyanate (KSCN) poisoning tests (Supplementary Figure S6A) were carried out to confirm the important role of Co sites, and the results showed that obvious recession occurred when 0.1 M KSCN solution was added in the acid electrolyte. The significant increase of the overpotential after KSCN treatment confirmed that Co species were the catalytic active sites (Li et al., 2020b). The Co-N-C_0.025M_ on glass carbon electrode had the same onset potential compared with RDE, which means an excellent intrinsic HER activity of Co-N-C_0.025M_, but a decreased current density was possibly caused by the mass transfer restriction (Supplementary Figure S6B). Moreover, Tafel slopes of these electrocatalysts were calculated (Figures 4C,D) so as to investigate the mechanism of HER activity. The lower Tafel slope of the Co-N-C_X_ catalysts revealed that the catalysts without acid etching possessed faster HER catalytic kinetics. Interestingly, neither of the Co-N-C_0.0125M_ catalyst mainly based on single-atom Co sites and the Co-N-C_0.075M_ catalyst with the largest Co NPs yielded the best HER activity. Both of the overpotential and Tafel slope of Co-N-C_0.025M_ were lowest among the Co-N-C_X_ and H-Co-N-C_X_ catalysts, representing the best HER catalytic activity. Meanwhile, the larger concentration of cobalt precursor catalysts possess recession HER performance, further confirming that the optimal MSI leads to a moderate hydrogen adsorption energy and improved electroactive sites (Du et al., 2020b). Furthermore, acid treatment could change the MSI, and the HER activity was suppressed. However, the catalytic performance of H-Co-N-C_0.05M_ was even a little better after acid etching, which might be due to the fact that the acid treatment etched off some larger Co NPs and made the MSI more appropriable for HER; this viewpoint can be proved by the below electrochemical surface area measurement.

To further verify the HER catalytic performance of various samples, cyclic voltammetry (CV) measurements with varying scan rates (see Supplementary Figure S7) were performed to explore the electrochemical double-layer capacitance (C_dl_) of as-prepared catalysts. The C_dl_ values were calculated to make an estimate of the electrochemical surface area (ECSA) of the as-synthesized catalysts (Figures 4E,F). Compared to other Co-N-C_X_ and N-C, Co-N-C_0.025M_ exhibited the largest C_dl_, suggesting that the Co-N-C_0.025M_ had more electroactive species. In addition, corresponding H-Co-N-C_X_ had larger ECSA but poorer performance than Co-N-C_X_, attributed to the changes of carbon stacking structure and MSI. Therefore, compared with the ECSA, the MSI was the key factor in determining the catalytic performance. Thus, the best HER activity for Co-N-C_0.025M_ is beneficial from the optimizing MSI due to the coexistence of single-atom Co sites and Co NPs.

**FIGURE 5:**
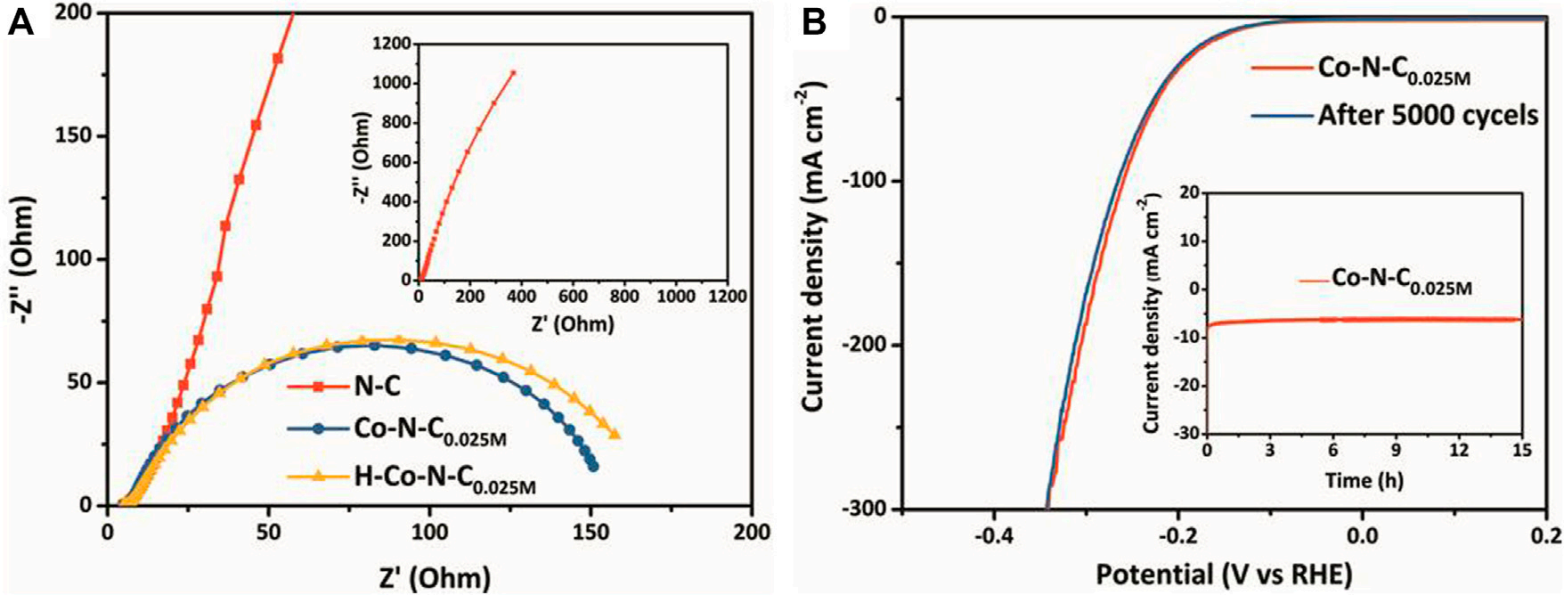
**(A)** Nyquist plots of Co-N-C_0.025M_, H-Co-N-C_0.025M_, and N-C. **(B)** LSV curves of Co-N-C_0.025M_ original and after 5,000 cycles; inset: time-dependent current density curve of Co-N-C_0.025M_ in 0.5 M H_2_SO_4_ under an overpotential of 145 mV.

The HER catalytic kinetics was further investigated by electrochemical impedance spectroscopy (EIS). The Nyquist and Bode (Supplementary Figure S8) plots of Co-N-C_0.025M_, H-Co-N-C_0.025M_, and N-C by applying an AC voltage with varying frequencies (range from 0.1 MHz to 0.1 Hz; amplitude 5 mV) were recorded at −0.215 V vs. RHE in 0.5 M H_2_SO_4_. As shown in Figure 5A, the H-Co-N-C_0.025M_ exhibited larger charge transfer resistance because of the larger arc radius in the high-frequency region than Co-N-C_0.025M_, which was attributed to the change in MSI caused by acid treatment. In addition, the similar diffusion resistance for the H-Co-N-C_0.025M_ and Co-N-C_0.025M_ in the low-frequency region confirms that the use of rotating disk electrode effectively reduces the resistance of mass transfer process. Both the resistances of Co-N-C_0.025M_ and H-Co-N-C_0.025M_ were much smaller than that of N-C, demonstrating that cobalt species acted as active sites and interacted with the nitrogen-doped carbon supports, which was beneficial to the adsorption of reactants and would speed up the kinetic process of HER; the Tafel slopes also proved this conclusion. Furthermore, a long-term hydrogen evolution test was performed to investigate the durability of Co-N-C_0.025M_. There was neither obvious degradation in HER activity after 5,000 cycles (Figure 5B) nor a significant decrease in current density after 15 h continuously working at −145 mV vs. RHE (inset of Figure 5B), suggesting that the stability of the Co-N-C_0.025M_ catalyst was remarkable.

## Conclusion

In summary, the Co-N-C_X_ catalysts with Co NPs encapsulated in nitrogen-doped graphitic carbon nanosheets were successfully synthesized via an organic–inorganic hybridization method. The concentration of cobalt precursor imposed a strong effect on the nanoparticle diameter and MSI of the Co-N-C_X_ catalysts. Meanwhile, encapsulated Co NPs affected the features of the surrounding carbon supports by means of altering the electron density and promoting electron transfer from the carbon supports to embedded Co NPs, thus generating a great synergic effect between encapsulated Co NPs and single-atom Co sites to improve electrocatalytic HER activity. The Co-N-C_0.025M_ catalyst without acid etching showed excellent catalytic performance for HER in acid medium, which was ascribed to its composite structure comprising single-atom Co sites and encapsulated Co NPs that optimally interact with surrounding carbon supports. This work may provide a potential approach for the design and preparation of high activity non-precious metal hybrid catalysts for electrocatalytic HER.

## Data Availability

The original contributions presented in the study are included in the article/Supplementary Material, further inquiries can be directed to the corresponding authors.
