# Supplementary material for: Impacts of Metal–Support Interaction on Hydrogen Evolution Reaction of Cobalt-Nitride-Carbide Catalyst
Source: Front Chem. 2022 Feb 1;9:828964. doi: 10.3389/fchem.2021.828964 (PMC8844497; doi:10.3389/fchem.2021.828964)
Supplement: Supplementary file 1 [file DataSheet1.docx]

Supplementary Material

Impacts of Metal-Support Interaction on Hydrogen Evolution Reaction of Cobalt-Nitride-Carbide Catalyst

Xuan Zhang^†^, Yu-An Li^†^, Yaozhen Huang, Haiqiang Mu, Xiaofeng Gu, Feng Li^*^, Zheng Wang^*^ and Jing Li^*^

State Key Laboratory of High-efficiency Utilization of Coal and Green Chemical Engineering, School of Chemistry and Chemical Engineering, Ningxia University, Yinchuan, China

*** Correspondence:**Feng Li, fengli@nxu.edu.cn; Zheng Wang, wzheng@nxu.edu.cn; Jing Li, jingli18@nxu.edu.cn

# Experimental Section

## Materials

D(+)-Glucose monohydrate (C_6_H_12_O_6_∙H_2_O), nickel nitrate hexahydrate (Ni(NO_3_)_2_∙6H_2_O), iron nitrate nonahydrate (Fe(NO_3_)_3_∙9H_2_O), copper nitrate trihydrate (Cu(NO_3_)_2_∙3H_2_O), sulfuric acid (H_2_SO_4_), hydrochloric acid (HCl) and absolute ethanol were purchased from Sinopharm Chemical Reagent Co. Ltd. (Shanghai, China). Dicyandiamide (C_2_H_4_N_4_), cobalt nitrate hexahydrate (Co(NO_3_)_2_∙6H_2_O), ammonium metatungstate hydrate ((NH_4_)_6_H_2_W_12_O_40_∙xH_2_O) and ammonium molybdate ((NH_4_)_2_MoO_4_) were purchased from Shanghai Macklin Biochemical Co. Ltd. (Shanghai, China). Nafion solution (5%) was purchased from Dupont Co. (USA). High pure hydrogen gas (99.999%) was purchased from Jinghua (Yinchuan) Gas Co., Ltd. (Yinchuan, China). All the chemicals were used without further purification.

## Characterization

Transmission electron microscopy (TEM) was performed on FEI Tecnai G2 20 microscope (FEI, Hillsboro, OR, USA) linked to an X-ray energy dispersive spectrometer (EDS). Aberration-corrected high angle annular dark field-scanning transmission electron microscopy (HAADF-STEM) was performed on Titan Themes Cubed G2 300 microscope (FEI, Hillsboro, OR, USA). X-ray powder diffraction (XRD) patterns were acquired from Bruker D8 Advance A25 diffractometer (Bruker, Karlsruhe, Germany) with Cu Ka radiation (k = 0.15418 nm). Raman spectroscopy measurement was performed on a DXR 2xi Raman microscope imaging spectrometer with a 532 nm excitation laser (Thermo Fisher Scientific, Waltham, UK). X-ray photoelectron spectroscopy (XPS) spectra were obtained using an ESCALAB 250 spectrometer (Thermo Fisher Scientific, Waltham, UK). The tapping mode atomic force microscope (AFM) images of Co-N-C_X_ nanosheets deposited on silicon substrates were taken using a Being CSPM 5500 Scanning Probe Microscope (Being, Guangzhou, China).

# Supplementary Figures and Tables

## Supplementary Figures


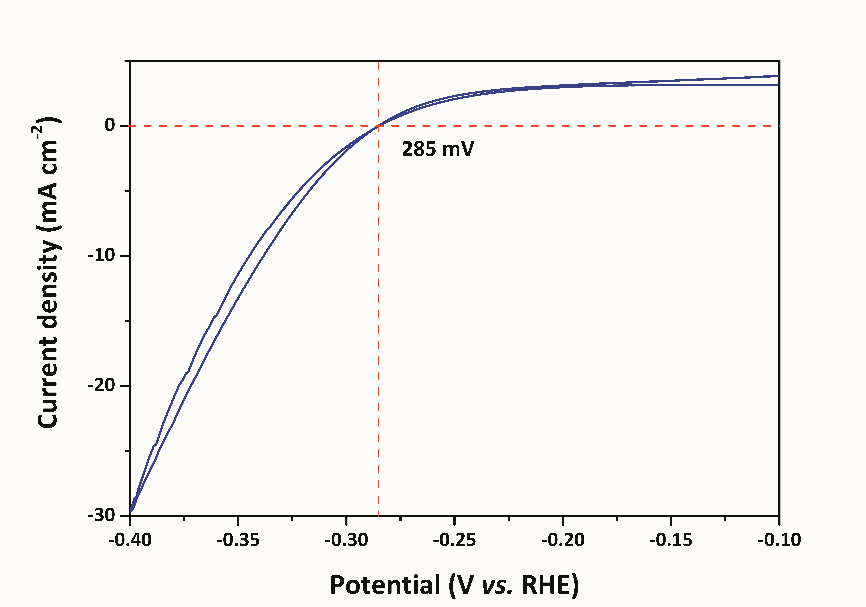


**Supplementary Figure 1.** Calibration of Ag/AgCl reference electrode with respect to RHE in 0.5 M H_2_SO_4_ at 2 mV s^-1^ scan rate.


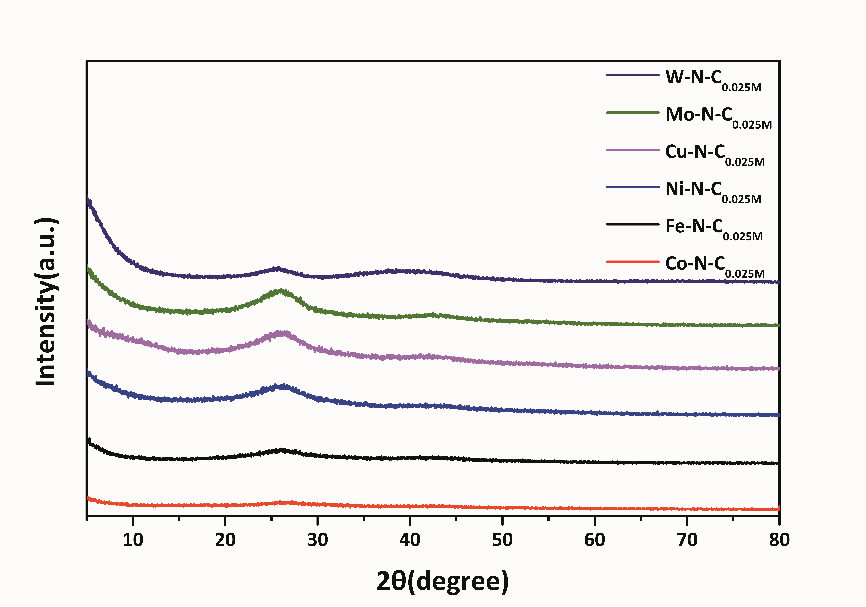


**Supplementary Figure 2.** XRD patterns of M-N-C_0.025M_.


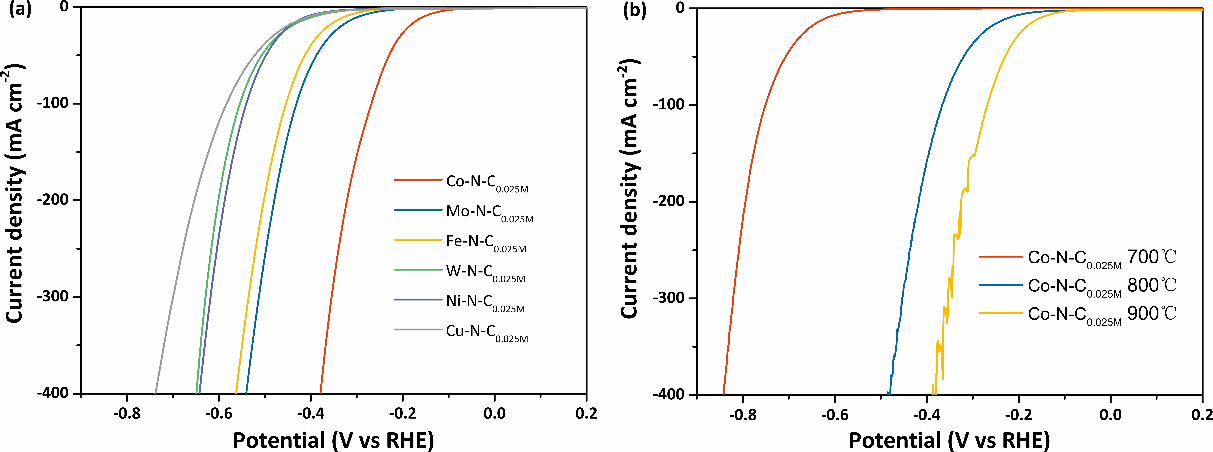


**Supplementary Figure 3.** (a) LSV curves of M-N-C_0.025M_. (c) LSV curves of Co-N-C_0.025M_ carbonized at 700 °C, 800 °C and 900 °C.


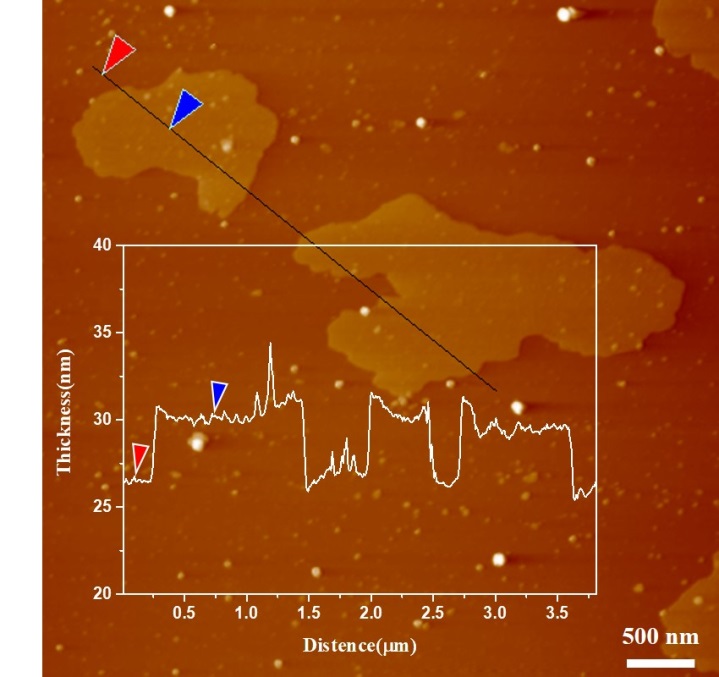


**Supplementary Figure 4.** AFM image of Co-N-C_0.025M_ and corresponding thickness analysis taken around the black line.


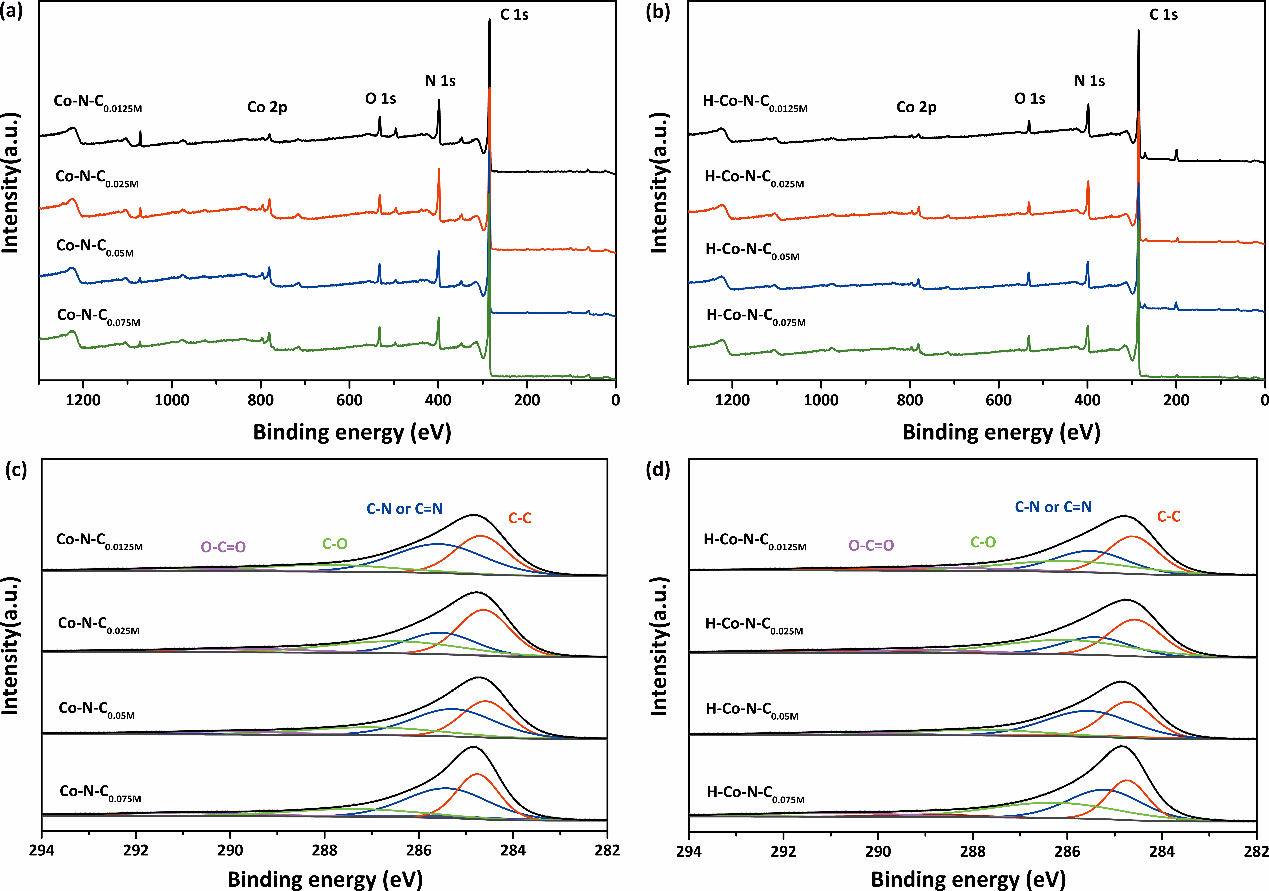


**Supplementary Figure 5.** XPS scans for (a) Co-N-C_X_ and (b) H-Co-N-C_X_, and high-resolution C 1s spectra of (c) Co-N-C_X_ and (d) H-Co-N-C_X_.


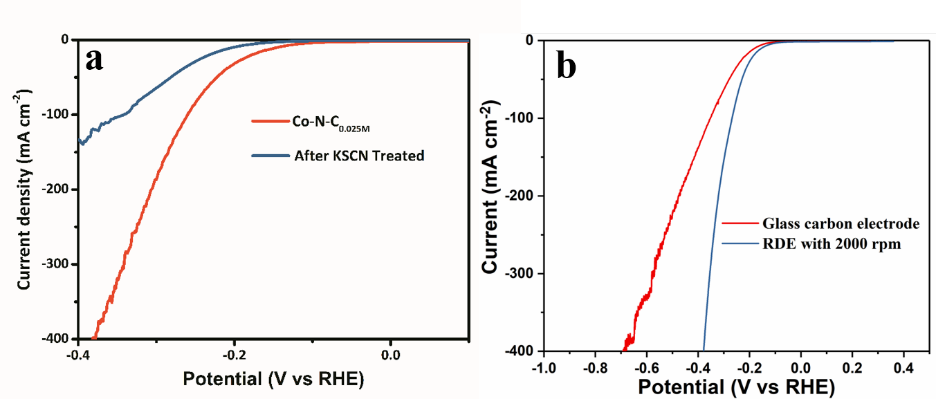


**Supplementary Figure 6.** (a) LSV curves of Co-N-C_0.025M_ before and after KSCN treatment, (b) comparative LSV curves of Co-N-C_0.025M_ on GCE and RDE.

**
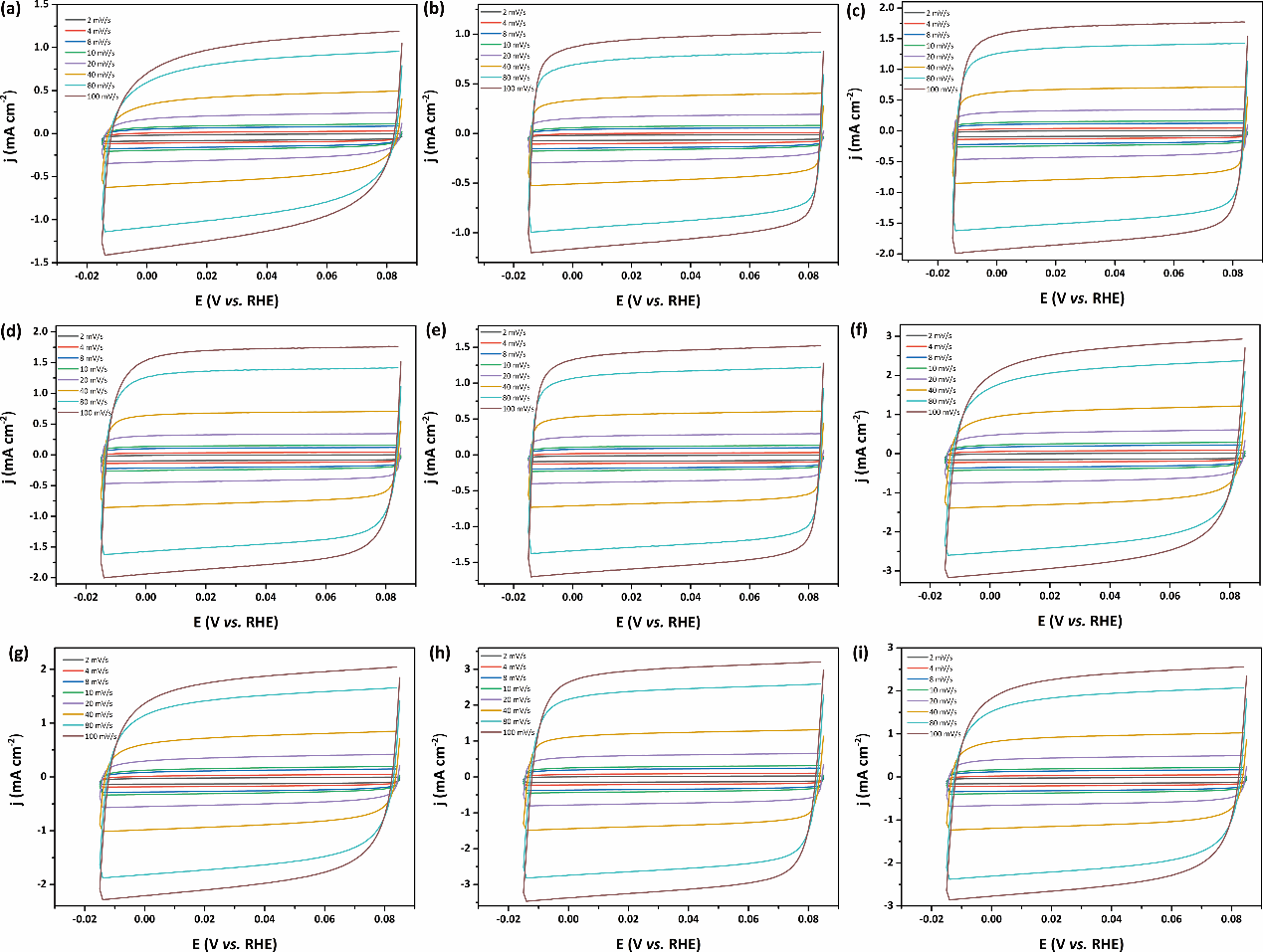
**

**Supplementary Figure 7.** Cyclic voltammetry measurements with varying scan rate of (a) N-C, (b) Co-N-C_0.0125M_, (c) Co-N-C_0.025M_, (d) Co-N-C_0.05M_, (e) Co-N-C_0.075M_, (f) H-Co-N-C_0.0125M_, (g) H-Co-N-C_0.025M_, (h) H-Co-N-C_0.05M_ and (i) H-Co-N-C_0.075M_.


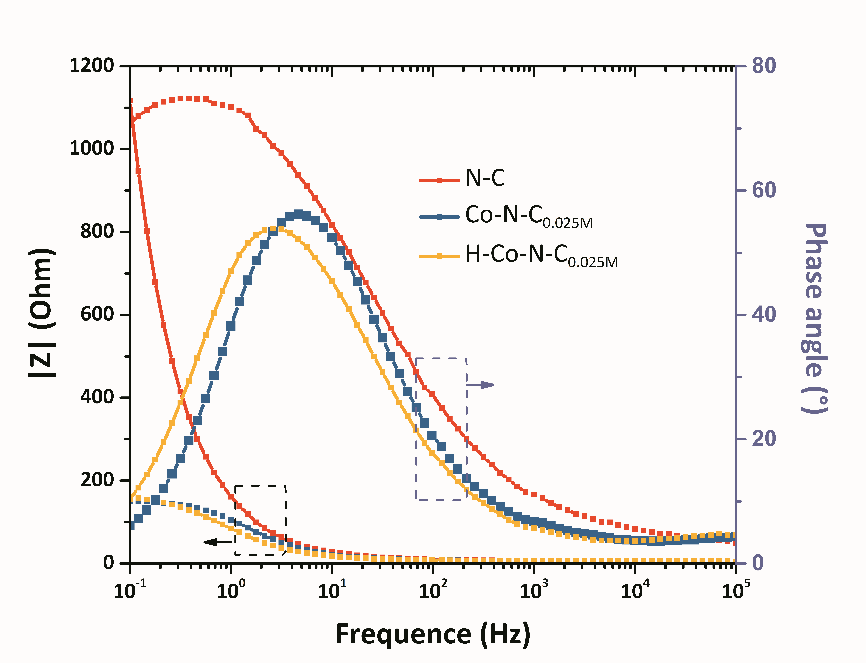


**Supplementary Figure 8.** Bode plots of Co-N-C_0.025M_, H-Co-N-C_0.025M_ and N-C.

## Supplementary Tables

**Supplementary Table 1.** Comparison of HER performance in 0.5 M H_2_SO_4_ between Co-N-C_0.025M_ and other electrocatalysts in literature.

| Catalysts | η@10 mA cm^-2^  (mV) | Tafel slope  (mV dec^-1^) | Catalysts loading  (mg cm^-2^) | References |
| --- | --- | --- | --- | --- |
| Co-C-N | 138 | 55 | 2 | Wang, et al. 2015 |
| Co@NC/NG | 200 (η_13.6_) | 79.3 | 0.285 | Zhou, et al. 2015 |
| Co@NG | 183 | 100 | 1.08 | Min, et al. 2000 |
| N-Co@G | 265 | 98 | 0.285 | Fei, et al. 2015 |
| N/Co-doped PCP//NRGO | 229 | 126 | 0.36^*^ | Hou, et al. 2015 |
| Co@N-CNTs@rGO | 87 | 52 | 0.5 | Chen, et al. 2018 |
| 4.55% Co-N/C | 168~180 | 108~106 | 0.78 | Du, et al. 2020 |
| Co-NG-5010-10 | 146 | 64 | 0.302 | Zhang, et al. 2018 |
| MFCo800 | 161 | 88.1 | 0.43 | Tang, et al. 2018 |
| Co@NCNTs-800 | 280 | 95.9 | 0.14 | Li, et al. 2017 |
| Co@NC-G-700 | 140 | 62 | 0.21 | Wen, et al. 2019 |
| MCo1.0 | 171 | 123.5 | 0.43 | Sun, et al. 2018 |
| Co-MOF-800 | 200 | 77 | 0.71^*^ | Pan, et al. 2019 |
| Co-N-C_0.025M_ | 145 | 74.13 | 0.24 | This work |

^*^Calculated from data reported in the literature.

Supplementary References

Wang, Z.-L.,Hao, X.-F.,Jiang, Z.,Sun, X.-P.,Xu, D.,Wang, J.,Zhong, H.-X.,Meng, F.-L.,Zhang, X.-B. (2015) C and N Hybrid Coordination Derived Co–C–N Complex as a Highly Efficient Electrocatalyst for Hydrogen Evolution Reaction. Journal of the American Chemical Society 137 (48), 15070-15073, doi: 10.1021/jacs.5b09021.

Zhou, W.,Zhou, J.,Zhou, Y.,Lu, J.,Zhou, K.,Yang, L.,Tang, Z.,Li, L.,Chen, S. (2015) N-Doped Carbon-Wrapped Cobalt Nanoparticles on N-Doped Graphene Nanosheets for High-Efficiency Hydrogen Production. Chemistry of Materials 27, 2026-2032, doi: 10.1021/acs.chemmater.5b00331.

Min, Z.,1, Y. L.,1, F. Z.,1, K. N.,Na, H.,1, X. W.,1, W. H.,1, X. S.,1, J. Z.,Mater., Y. L. J. K. L. f. C.-B. F.,Devices. (2000) Metallic Cobalt Nanoparticles Encapsulated in Nitrogen-Enriched Graphene Shells: Its Bifunctional Electrocatalysis and Application in Zinc–Air Batteries. Advanced Functional Materials, doi: 10.1002/adfm.201600636.

Fei, H.,Yang, Y.,Peng, Z.,Ruan, G.,Zhong, Q.,Li, L.,Samuel, E. L. G.,Tour, J. M. (2015) Cobalt Nanoparticles Embedded in Nitrogen-Doped Carbon for the Hydrogen Evolution Reaction. ACS Applied Materials & Interfaces 7 (15), 8083-8087, doi: 10.1021/acsami.5b00652.

Hou, Y.,Wen, Z.,Cui, S.,Ci, S.,Mao, S.,Chen, J. (2015) An Advanced Nitrogen‐Doped Graphene/Cobalt‐Embedded Porous Carbon Polyhedron Hybrid for Efficient Catalysis of Oxygen Reduction and Water Splitting. Advanced Functional Materials 25 (6), 872-882, doi: 10.1002/adfm.201403657

Chen, Z.,Wu, R.,Liu, Y.,Ha, Y.,Guo, Y.,Sun, D.,Liu, M.,Fang, F. (2018) Ultrafine Co Nanoparticles Encapsulated in Carbon‐Nanotubes‐Grafted Graphene Sheets as Advanced Electrocatalysts for the Hydrogen Evolution Reaction. Advanced Materials 30 (30), 1802011.1-1802011.10, doi: 10.1002/adma.201802011

Du, Y.,Chen, H.,Huang, Z.,He, X.,Fang, W.,Li, W.,Zhao, L. (2020) Unique Size of Co Nanoparticles Encapsulated in N-Rich Carbon Structure Derived from Electrochemical Etching for Enhanced Electrocatalytic Hydrogen Evolution. ACS Applied Energy Materials 3 (1), 687-694, doi: 10.1021/acsaem.9b01877.

Zhang, Y.,Li, W.,Lu, L.,Song, W.,Wang, C.,Zhou, L.,Liu, J.,Chen, Y.,Jin, H.,Zhang, Y. (2018) Tuning active sites on cobalt/nitrogen doped graphene for electrocatalytic hydrogen and oxygen evolution. Electrochimica Acta 265, 497-506, doi: 10.1016/j.electacta.2018.01.203.

Tang, D.,Li, K.,Zhang, W.,Qiao, Z.-A.,Zhu, J.,Zhao, Z. (2018) Nitrogen-doped mesoporous carbon-armored cobalt nanoparticles as efficient hydrogen evolving electrocatalysts. Journal of Colloid and Interface Science 514, 281-288, doi: 10.1016/j.jcis.2017.12.026 .

Li, J.-S.,Du, B.,Lu, Z.-H.,Meng, Q.-T.,Sha, J. (2017) In situ-generated Co@nitrogen-doped carbon nanotubes derived from MOFs for efficient hydrogen evolution in both alkaline and acidic conditions. New J. Chem. 41, doi: 10.1039/C7NJ02334K.

Wen, X.,Yang, X.,Li, M.,Bai, L.,Guan, J. (2019) Co/CoOx nanoparticles inlaid onto nitrogen-doped carbon-graphene as a trifunctional electrocatalyst. Electrochimica Acta 296, 830-841, doi: 10.1016/j.electacta.2018.11.129.

Sun, X.,Zhang, W.,Tang, D.,Qiao, Z.-A.,Liu, Y.,Liang, D.,Zhu, J.,Zhao, Z. (2018) Co-entrapped, N-doped mesoporous carbons prepared from melamine formaldehyde resins with CoCl2 as template for hydrogen evolution. *Journal of Colloid and Interface Science* 516, 416-422, doi: 10.1016/j.jcis.2018.01.091.

Pan, Z.,Pan, N.,Chen, L.,He, J.,Zhang, M. (2019) Flower-like MOF-derived Co–N-doped carbon composite with remarkable activity and durability for electrochemical hydrogen evolution reaction. *International Journal of Hydrogen Energy* 44 (57), 30075-30083, doi 10.1016/j.ijhydene.2019.09.117.
